# First Draft Assembly and Annotation of the Genome of a California Endemic Oak *Quercus lobata* Née (Fagaceae)

**DOI:** 10.1534/g3.116.030411

**Published:** 2016-09-12

**Authors:** Victoria L. Sork, Sorel T. Fitz-Gibbon, Daniela Puiu, Marc Crepeau, Paul F. Gugger, Rachel Sherman, Kristian Stevens, Charles H. Langley, Matteo Pellegrini, Steven L. Salzberg

**Affiliations:** * Department of Ecology and Evolutionary Biology, University of California, Los Angeles, California 90095; † Institute of the Environment and Sustainability, University of California, Los Angeles, California 90095; ‡ Institute of Genomics and Proteomics, University of California, Los Angeles, California 90095; § Center for Computational Biology, McKusick-Nathans Institute of Genetic Medicine, Johns Hopkins University School of Medicine, Baltimore, Maryland 21205; ** Department of Evolution and Ecology, University of California, Davis, California 95616; †† University of Maryland Center for Environmental Science, Appalachian Laboratory, Frostburg, Maryland 21532; ‡‡ Department of Computer Science, Johns Hopkins University, Baltimore, Maryland 21218; §§ Department of Molecular, Cell, and Developmental Biology, University of California, Los Angeles, California 90095; *** Department of Biomedical Engineering, Johns Hopkins University, Baltimore, Maryland 21218; ††† Department of Biostatistics, Johns Hopkins University, Baltimore, Maryland 21218

**Keywords:** adaptation, annotation, chloroplast, nuclear genome assembly, *Quercus*, GenPred, Shared Data Resources, Genomic Selection

## Abstract

Oak represents a valuable natural resource across Northern Hemisphere ecosystems, attracting a large research community studying its genetics, ecology, conservation, and management. Here we introduce a draft genome assembly of valley oak (*Quercus lobata*) using Illumina sequencing of adult leaf tissue of a tree found in an accessible, well-studied, natural southern California population. Our assembly includes a nuclear genome and a complete chloroplast genome, along with annotation of encoded genes. The assembly contains 94,394 scaffolds, totaling 1.17 Gb with 18,512 scaffolds of length 2 kb or longer, with a total length of 1.15 Gb, and a N50 scaffold size of 278,077 kb. The *k*-mer histograms indicate an diploid genome size of ∼720–730 Mb, which is smaller than the total length due to high heterozygosity, estimated at 1.25%. A comparison with a recently published European oak (*Q. robur*) nuclear sequence indicates 93% similarity. The *Q. lobata* chloroplast genome has 99% identity with another North American oak, *Q. rubra*. Preliminary annotation yielded an estimate of 61,773 predicted protein-coding genes, of which 71% had similarity to known protein domains. We searched 956 Benchmarking Universal Single-Copy Orthologs, and found 863 complete orthologs, of which 450 were present in > 1 copy. We also examined an earlier version (v0.5) where duplicate haplotypes were removed to discover variants. These additional sources indicate that the predicted gene count in Version 1.0 is overestimated by 37–52%. Nonetheless, this first draft valley oak genome assembly represents a high-quality, well-annotated genome that provides a tool for forest restoration and management practices.

The genus *Quercus* (oak) is one of the most important trees in the Northern Hemisphere and, based on USDA Forest Service data, oaks make up the largest proportion of total biomass and have the highest number of species of the five major tree genera in the United States (Cavender-Bares 2016). Oaks are valuable economically, and, in North America, *Quercus* is a major source of hardwood lumber, especially for furniture, railroad ties, pallets, flooring, and export (Luppold and Bumgardner 2013). In 2008, oaks accounted for over 38% of hardwood lumber production in the United States, in an industry that sold 1.8 billion board feet that year (Espinoza *et al.* 2011). In 2011, over 1,066,100 m^3^ of oak lumber, and 451,000 m^3^ of oak logs, were exported from the United States (Luppold and Bumgardner 2013). In California, oaks offer a number of ecosystem services with tangible economic benefits. Oaks add economic value to hunting lands and rangelands (Standiford and Howitt 1993; Kroeger *et al.* 2010) by improving nutrient composition and cycling (Dahlgren *et al.* 1997; Herman *et al.* 2003). Riparian oaks, such as valley oak (*Quercus lobata*), are important in stabilizing soil, maintaining aquatic and terrestrial animal habitat, reducing pesticide runoff, and improving water quality (Dosskey *et al.* 1997; Kroeger *et al.* 2010). Furthermore, the presence of oaks has been shown to support exceptionally diverse native plant, bird (Howard 1992), vertebrate (Block *et al.* 1990), and arthropod communities (Swiecki *et al.* 1997). Oaks are culturally significant in California as iconic parts of vineyards, pastoral landscapes, residential developments, and, in particular, an historically important source of food for native cultures (Pavlik *et al.* 1991; Anderson 2005). Thus, the management and maintenance of oak ecosystems is of high priority.

Oaks are managed in natural stands with selective harvesting accompanied by natural regeneration, or, especially in Europe (*e.g.*, von Lupke 1998), in plantations. North American forestry has a long tradition of planting seeds locally under the assumption that trees are locally adapted. However, one question is how local is local (McKay *et al.* 2005), especially when we may need to manage tree populations differently under current conditions of rapid climate change (Spittlehouse and Stewart 2004; Millar *et al.* 2007; Aitken and Whitlock 2013; Aitken *et al.* 2008). Not only were many of our current oak populations established under past climates that differ from today’s, but current locations may experience warmer climates in the next 50 yr (*e.g.*, Sork *et al.* 2010). Thus, among the many concerns of restoration ecologists in restoring ecosystems is the source of seeds used to replant an oak stand, given that the future climate of that site may be different from the current one (Aitken and Whitlock 2013). An understanding of the genes that underlie adaptation to climate would inform selection of seed sources for oak management to yield resilient forests for future climate conditions.

Given the significance of *Quercus* species to ecosystems across the Northern Hemisphere, and basic and applied questions in population genetics, evolutionary ecology, conservation science, and forest management are increasingly using genomic approaches (Petit *et al.* 2013). This year, a draft genome of *Q. robur*, a common western European oak in the section *Quercus*, assembled with Sanger, Roche 454, and Illumina-generated sequences, became publicly available (Plomion *et al.* 2016). Last year, two reference transcriptome assemblies were published—one for the California endemic oaks, *Q. lobata* and *Q. garryana* (Cokus *et al.* 2015), and another for the European oaks, *Q. robur* and *Q. petraea* (Lesur *et al.* 2015). These sets of species come from the same white oak section (*Quercus* sect. *Quercus*), but they differ markedly in recent evolutionary history. The European oaks experienced severe bottlenecks during Pleistocene glaciations, with most populations surviving in southern refugia (Petit *et al.* 2003; Petit *et al.* 2002), while the California endemic oaks have not experienced serious bottlenecks in at least 150,000 yr or more, with evidence of relatively stable species distributions during the last few interglacial cycles (Gugger *et al.* 2013). Many oak studies have utilized genomic approaches, but without the benefit of a reference genome (*e.g.*, Derory *et al.* 2006; Gugger *et al.* 2016a; Spiess *et al.* 2012; Sork *et al.* 2016). The ability to identify reliable gene models, and to understand genome evolution, requires a high-quality oak genome with high accuracy, short-range contiguity, long-range connectivity, and good coverage.

Here, we introduce a draft genome of the nuclear and chloroplast DNA sequences of a California endemic oak species, *Q. lobata*, as a first step toward producing an even higher quality genome with in-depth annotations. We selected a target *Q. lobata* adult (#786, 34.68820°, −120.03615°, Figure 1) from a population of valley oaks at the UC-Santa Barbara Sedgwick Nature Reserve that is publicly available through the University of California Nature Reserve System. This particular tree was chosen because it is a prolific acorn producer, and is part of ongoing research of valley oak, such as a 95-locality and 6000 tree-based provenance study of valley oak (Delfino-Mix *et al.* 2015), epigenetic and landscape genomic studies (Gugger *et al.* 2016b; Sork *et al.* 2016), contemporary gene flow studies (Grivet *et al.* 2009), and a multi-year phenology study (A. Lentz and V. L. Sork, unpublished data). This study has five goals: (1) introduce a draft genome assembly of the valley oak genome (version 1.0); (2) present a first set of annotations, taking advantage of annotations we developed while producing a reference transcriptome for *Q. lobata* (Cokus *et al.* 2015); (3) report on an earlier draft of our genome (version 0.5) that is less complete for gene models, but has excluded duplicated haplotypes to facilitate identification of nucleotide variants; (4) summarize publicly available genomic resources developed to date; (5) compare our findings with the European oak *Q. robur* (Plomion *et al.* 2016). This valley oak genome assembly here represents the first phase of the production of a higher-quality, well-annotated genome that will serve as a valuable resource for the oak genetic and conservation communities.

**Figure 1:**
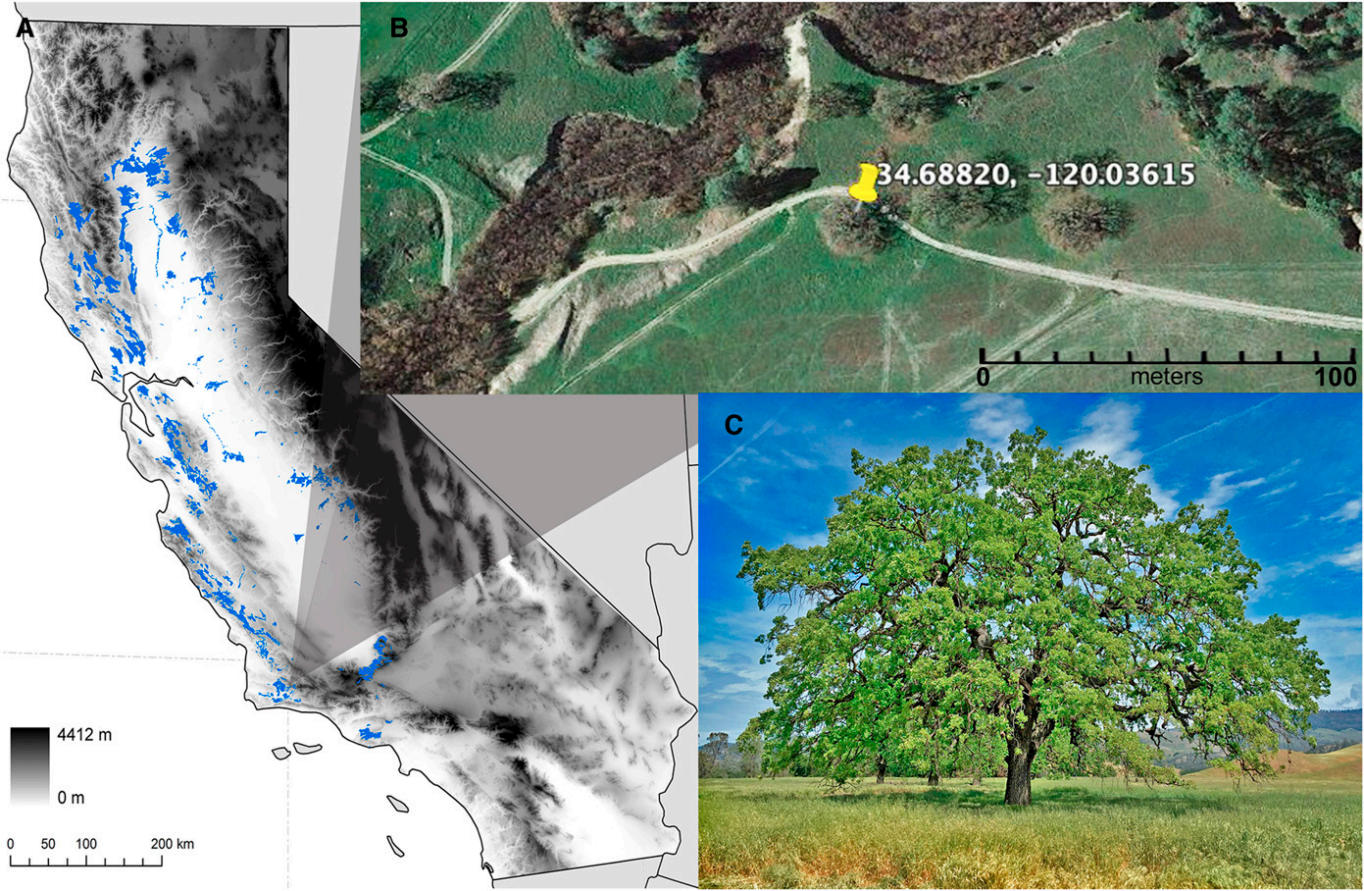
Information on sequenced *Q. lobata* adult #786. (A) Map of California with species distribution indicated in blue, and location of sequenced tree shown with shaded triangle. (B) Local map of sequenced tree #786 within the University of California Santa Barbara Sedgwick Reserve in the San Ynez Valley, Santa Barbara Co., CA. (C) Photo of the sequenced tree *#7*86. (Photo by A. Lentz.)

## Materials and Methods

### DNA isolation

Leaves were collected in September 2014, flash-frozen with liquid nitrogen, and stored at −80° prior to DNA extraction. Nuclei were extracted from 40 g of leaves as described in Zimin *et al.* (2014). Nuclei were lysed by the addition of *N*-laurylsarcosine to a final concentration of 1% (w/v), and incubation at room temperature for 15 min. Sodium chloride was added to a final concentration of 0.7 M, then hexadecyltrimethylammonium bromide (CTAB) to a final concentration of 1% (w/v), and the mixture was incubated 1 hr at 60°. After two consecutive extractions with equal volumes of 24:1 chlorform:isoamyl-alcohol, the DNA was precipitated by the addition of 2/3 volume isopropanol, removed from solution with a glass hook, and resuspended in TE buffer. The DNA was then incubated for 1 hr at 37° with DNase-free RNase, extracted once with 25:24:1 phenol:chloroform:isoamyl-alcohol, then once with 24:1 chlorform:isoamyl-alcohol, ethanol precipitated, and resuspended in TE buffer. A final purification of the DNA was performed using the Genomic DNA Clean and Concentrator kit (Zymo Research) following the manufacturer’s instructions. Purified DNA was then treated with 1 µl of PreCR Repair Mix (New England Biolabs) per 3 µg of DNA following the manufacturer’s instructions, extracted once with 25:24:1 phenol:chloroform:isoamyl-alcohol, then once with 24:1 chlorform:isoamyl-alcohol, ethanol precipitated, and resuspended in TE buffer. The purified and repaired DNA was quantified on a NanoDrop spectrophotometer (Thermo Fisher Scientific).

### Construction of paired-end libraries

Two short-insert paired-end libraries were constructed, one with PCR enrichment, and one without. To create the PCR-enriched library, 5 µg of DNA was end-repaired and A-tailed, and then universal Illumina paired-end adapters were ligated. The ligation product was run on a 2% agarose size-selection gel, and a ∼1 mm thick slice was cut from the gel adjacent to the 600 bp ladder marker. The DNA was extracted from the gel material using a MinElute kit (Qiagen), and quantified on a Bioanalyzer 2100 (Agilent Technologies); 7.6 ng of DNA was used as template for 10 cycles of PCR in a 50 µl reaction volume using KAPA HiFi HotStart ReadyMix (Kapa Biosystems) and barcoded Illumina primers. The PCR-free library was constructed using the TruSeq DNA PCR-free LT kit (Illumina) following the manufacturer’s instructions for 550 bp inserts.

### Long-insert mate pair (jumping) libraries

Long-insert mate pair libraries were constructed using the Nextera Mate Pair Sample Prep kit (Illumina) following the manufacturer’s “Gel-Plus” instructions with the following modifications: the products of three tagmentation/strand-displacement reactions were pooled for size-selection; all size selections were performed in 0.6% MegaBase agarose (Bio-Rad) gels electrophoresed using a buffer-recirculating pump, and a FIGE Mapper (Bio-Rad) as follows: 1× TAE buffer; 16 hr run at room temperature; 4.1 V/cm forward, and 2.7 V/cm reverse field strength, both with linear ramping from 0.1 sec initial to 0.8 sec final switch time; finally, the purified DNA fractions from the size-selection gel were loaded directly onto a fresh 0.6% MegaBase agarose gel, and size-selected a second time to increase the stringency of the size-selection prior to fragment circularization.

### Sequence data

All sequencing was performed on three lanes of a HiSeq2500 in Rapid Run mode with HCS version 2.2.58 and RTA version 1.18.64.0. The short-insert paired-end libraries were sequenced with read lengths of 250 bp, and the long-insert mate pair libraries were sequenced with read lengths of 150 bp. Demultiplexing and BCL to fastq conversion were performed with CASAVA version 1.8.2 (Eren *et al.* 2013).

### Assembly methods

We used the MaSuRCA assembler (Zimin *et al.* 2013) to assemble the short paired-end reads into super-reads. Input for this step was 266,002,352 read pairs (532,004,704 reads) from 500 and 550 bp fragments, with a read length of 250 bp. This represented a total of 133 Gb of sequence data, ∼175× coverage assuming a genome size of 750 Mb. We also used MaSuRCA to “clean” the reads from both short and long fragment libraries, in a process that uses super-reads to correct errors, and trim low-quality sequences from the ends of reads. We had nine long-fragment paired libraries, ranging in length from 2900 to 12,000 bp, with a total of 159,115,423 pairs of reads (∼56× coverage) in total. Subsequent assembly steps used SOAPdenovo2 (Luo *et al.* 2012) with the parameters −K 127 −k 63 to assemble super-reads into contigs and scaffolds, and to rescaffold.

To remove redundant and low-quality scaffolds, we aligned all scaffolds shorter than 50 kb to each other using bwa (Li and Durbin 2009) and MUMmer (Kurtz *et al.* 2004), and used the show-coords program within MUMmer to identify scaffolds that were completely contained by, and nearly identical to, other, longer, scaffolds. To align the *Q. lobata* and *Q. robur* genomes, we used both bwa-mem and MUMmer, which gave comparable results.

Previously, we had also created a haplotype-reduced version of the *Q. lobata* genome, labeled version 0.5 and available from http://valleyoak.ucla.edu. This version was created from an earlier draft assembly by aligning all *vs.* all contigs (> 600 bases) using BWA, and removing the smaller contig of any overlapping pair where at least 20% of the smallest contig is overlapping. This process was repeated until no more contigs were removed. There were 40,158 remaining contigs, totaling 760 Mb, average size 18,900 bp, with an N50 of 95,000 bp. This aggressive reduction of haplotype redundancy increases the utility of the genome for variant calling, albeit at the expense of creating gaps in the overall coverage.

As one assessment of the completeness of the genome, we conducted a BUSCO analysis (Simão *et al.* 2015). BUSCO (Benchmarking Universal Single-Copy Orthologs) software was used to search our genome for each of 956 plant orthologs within an early access version of the plants BUSCO dataset that had kindly been provided by the authors. BUSCO uses Augustus (Stanke *et al.* 2004) to predict gene models in targeted genomic regions, and was set to use precomputed metaparameters for Arabidopsis. For comparison, we also ran the same analysis on *Q. lobata* v0.5, the *Q. robur* genome, and the *Populus trichocarpa* genome (Tuskan *et al.* 2006).

To determine relative depths of coverage for BUSCO genes, the 133 million pairs of reads from our first library (Qlob_2_H00GJ_L001) were mapped to the *Q. lobata* v0.5 assembled contigs using bwa mem. GATK’s DepthOfCoverage (DePristo *et al.* 2011) was used to determine the mean coverage across each gene, for all mapped reads regardless of mapping quality.

### Chloroplast genome assembly

The chloroplast is present in multiple copies per cell, yielding much deeper read coverage than the chromosomal genome. This enables assembly from a relatively small fraction of the whole-genome data set. We extracted the first 10 million read pairs from one of the short fragment libraries (Qlob_2_H00GJ_L001), and used the ea-utils (Aronesty 2011) to trim adapters and low-quality sequence. We assembled the trimmed reads with SOAPdenovo2 (Luo *et al.* 2012) with parameters −K 127 −k 63, and then filled gaps within scaffolds using GapFiller (Boetzer and Pirovano 2012) with parameters −m 63 and −d 255. We aligned the resulting scaffolds to the NCBI chloroplast database (Sayers *et al.* 2012), and found seven that aligned to chloroplasts, with the best matches found in *Q. rubra* (northern red oak, NCBI accession NC_020152) (Alexander and Woeste 2014). Six of the seven scaffolds had > 30× coverage, and were longer than 500 bases, and we then aligned these six scaffolds to the *Q. rubra* chloroplast using nucmer (Kurtz *et al.* 2004). We noted that, while four of the scaffolds had consistent coverage depth of 30–40×, the other two had deeper coverage, 58× and 72×, indicating that some portions of these scaffolds represented duplicated regions. Consistent with this observation, the high-coverage scaffolds, C437218 and C437210, each aligned to two distinct locations in the *Q. rubra* chloroplast. We created duplicate copies of parts of these two scaffolds and added these to the six original scaffolds. The eight resulting scaffolds had overlapping ends, where all overlaps were of length 127 bp. Using *Q. rubra* as a guide, we constructed a gap-free circular arrangement of the scaffolds, and merged the overlapping ends to obtain the first version of the chloroplast genome.

We then aligned the original 10 million reads back to the assembly, and reviewed the depth of coverage with IGV (Robinson *et al.* 2011), which revealed three low-coverage regions. Two of these were mis-assemblies, in which contig ends had been concatenated where they should have been merged, creating an artificial tandem repeat. We corrected these by breaking the assembly, and manually merging the contig ends. This step resulted in even coverage across the two regions, and improved alignments with other oak chloroplast genomes. The third low coverage region corresponded to a 75-bp stretch of Ns that GapFiller had been unable to resolve. We then extracted all reads from the original 10 million that overlapped the edges of this gap, and assembled them using Minimus (Sommer *et al.* 2007). This step produced a single contig that spanned the gap. Merging this contig into the gap resulted in even-read coverage across this region, and produced the final, 161,289-bp chloroplast assembly.

To annotate the chloroplast assembly, genes from the *Q. rubra* chloroplast were downloaded from NCBI (accession NC_020152), and mapped to the final *Q. lobata* chloroplast using GMAP (Wu and Watanabe 2005). The resulting annotation contains 80 distinct genes, seven of which are duplicated once, and one twice for a total of 89 genes. There are 31 distinct tRNAs, and nine duplicates, resulting in a total of 40 tRNA genes. The annotated genic regions make up 60.95% of the chloroplast genome.

### Annotation

#### Gene models:

We used MAKER (Campbell *et al.* 2014) to identify gene models and predict functional annotations in version 1.0, and then transferred those annotations to version 0.5 using the default pipeline of FLO (https://github.com/wurmlab/flo), which is based on the University of California, Santa Cruz (UCSC)-Kent. Toolkit (Kuhn *et al.* 2012). MAKER was initially run in est2genome mode, which predicts genes directly from EST evidence. The EST evidence, which was based on our published transcriptome (Cokus *et al.* 2015), included the 28,261 transcripts for which gene models had previously been predicted. Additionally, protein evidence was provided as 324,038 protein sequences gathered from the genomes of seven plants: *Arabidopsis thaliana*, *P. trichocarpa*, *Vitis vinifera*, *Glycine max*, *Ricinus communis*, *Medicago truncatula*, and *Theobroma cacao*. RepeatMasker (Smit *et al.* 1996) was also run within the MAKER framework, using –species all. Transposable elements with nucleotide or protein level similarity to a set of 24,916 known transposons (provided by MAKER) were masked. Annotation was limited to contigs > 10 kb. The initial run was terminated after prediction of 1362 gene models. From these, 701 high confidence models were selected for training SNAP (Johnson *et al.* 2008). High confidence was determined by the maker2zff default minimums: 50% of splice sites confirmed by EST alignment, 50% of exons match an EST alignment, 50% of exons overlap any evidence (EST or Protein), and maximum annotation edit distance (AED) of 0.5. These 701 high confidence gene models were used to train SNAP—an *ab initio* gene predictor. A second round of MAKER was run using the HMMs from snap training for gene prediction, rather than the est2genome mode. All other settings were the same as for the first run, with the transcripts now being used only as evidence to support *ab initio* gene predictions. Two more rounds followed, resulting in 6710 and then 10,885 high confidence gene models. These 10,885 high confidence gene models were used to train the *ab initio* gene predictor in Augustus (Stanke *et al.* 2004). A final round of predictions were done using three gene predictors, SNAP and Augustus trained for this genome, and FGENESH (Salamov and Solovyev 2000) using the provided Dicot training set. This process resulted in our final set of 61,773 gene models, of which 13,898 are high confidence, as defined above.

#### Functional:

Protein sequences from our 61,773 predicted gene models were searched against the UniProt/Swiss-Prot database (The UniProt Consortium 2015; downloaded February 12, 2016; 550,299 entries), using Blastp with an *e*-value cutoff of 1 × 10^−6^ and seg masking. InterProScan was used to link our predicted proteins with gene ontology (GO; Ashburner *et al.* 2000) terms and Pfam (Finn *et al.* 2014) domains. These annotations are included in *Q. lobata* v1.0 gene models file (gff format). To quantify the presence of repetitive elements known to exist in plants, we ran RepeatMasker (version open-4.0.5, Smit, A. F. A. and P. Green, RepeatMasker, http://www.repeatmasker.org) against the eudicotyledons subset of RepBase (update 20150807).

### Data availability

Illumina sequence reads, genome assemblies, and annotation files for version 1.0 are available through NCBI BioProject PRJNA308314 and both versions with annotations are available at https://valleyoak.ucla.edu/genomicresources/.

## Results and Discussion

### Assembly results

The MaSuRCA assembly of paired reads created 13,129,188 super-reads, with an average length of 444 bp, totaling 5.84 Gb. Super-reads are highly accurate, and represent a very substantial compression of the original data, as they did here. Due to their greater length, they usually provide a better basis for assembly. These super-reads were then assembled into contigs and scaffolds using SOAPdenovo2 (Luo *et al.* 2012), with the parameters −k 127 −k 63. The resulting scaffolds contained 242,966 gaps, to which we applied the GapCloser from the SOAPdenovo package, which used the reads in a second pass to close ∼150 k gaps, leaving 96,589 gaps in an assembly with 806,091 scaffolds.

The scaffold cleaning procedure identified 605,682 redundant scaffolds, most of which were very short (average length 404 bp). These were removed, and we then rescaffolded the remaining 200,409 nonredundant scaffolds using SOAPdenovo2 with the same parameters as previously. This step resulted in 94,394 scaffolds, totaling 1.17 Gb in the final assembly. The assembly contains 18,512 scaffolds of length 2 kb or longer, with a total length of 1.15 Gb (Table 1).

**Table 1:** Summary statistics for assembly of *Quercus lobata*

	Number	Total size (bp)	N50 Size (bp)	Mean Size (bp)
All scaffolds	94,394	1,182,727,890	278,077	12,529
Scaffolds ≥ 2000 bp	18,512	1,153,710,009	278,077	62,322

N50 size defined as the value *N* such that at least 50% of the genome is covered by scaffolds of size *N* or larger. We used 730 Mb as the genome size for the N50 calculation.

### Genome size estimates

Independently of assembly, we characterized the genome sequence using the distribution of all short high-quality words of length *k* from the raw reads. This was done using *k*-mer histograms computed from the error-corrected reads using the program jellyfish (Marcais and Kingsford 2011), with word sizes (*k*) of 25 and 31.

**Figure 2:**
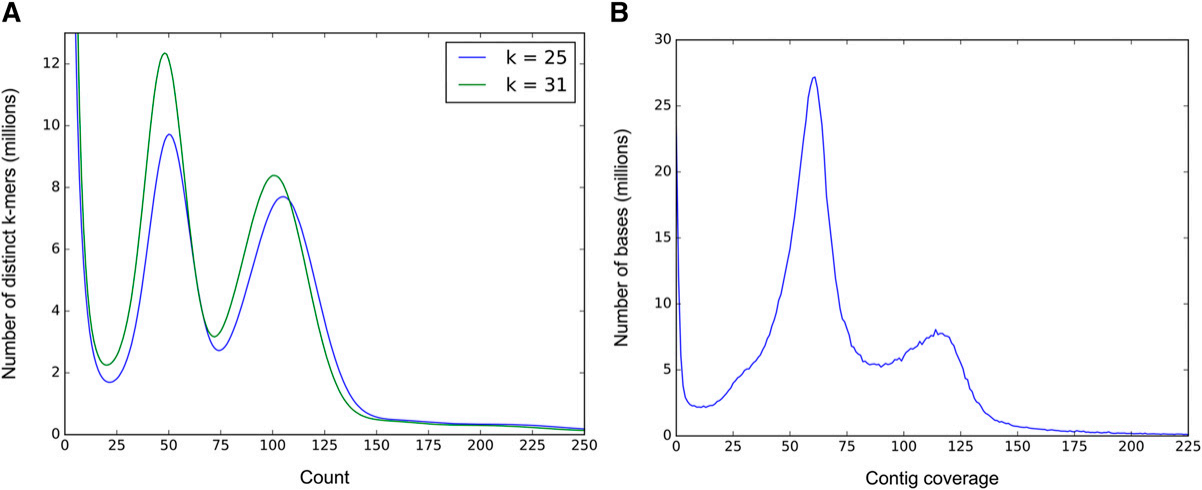
(A) Histograms of *k*-mer frequencies in the raw read data for *k* = 25 (blue) and *k* = 31 (green). The *x*-axis shows the number of times a *k*-mer occurred; *e.g.*, the peaks near *x* = 50 indicate the number of *k*-mers that occurred 50 times in the data. (B) Histogram of contig coverage in the assembly, based on mapping all reads back to the assembled contigs. The left peak shows the number of bases in contigs with 55–60× coverage, which correspond to regions where the assembler created two distinct contigs for divergent putative haplotypes. The right peak, at ∼110–120× coverage, contains contigs from regions where the genome are less variable, allowing the assembler to construct a single contig for those regions.

Both *k*-mer histograms display three distinct peaks (Figure 2, A and B). The extreme peak in Figure 2A at *k* = 1, representing ∼1% of the distinct *k*-mers, is an artifact caused by sequencing errors, each of which creates a *k*-mer that almost never occurs in the genome, and is therefore unique in the data set. The two peaks of interest, together comprising the largest area of each histogram, characterize the bimodal distribution that we expect from a heterozygous diploid genome. The right “diploid” peak, *k*-mers shared between homologous chromosomes, is consistently twice as deep as the left “haploid” peak, which contains *k*-mers unique to a haplotype due to heterozygosity (Table 2). For both *k* = 25 and *k* = 31, there were noticeably more words that were unique to a haplotype (*i.e.*, in the left peak) than words shared between them. This apparently high level of heterozygosity is consistent with values reported elsewhere for *Q*. *lobata* (Cokus *et al.* 2015), and expected for a highly outcrossing tree species. Both *k*-mer sizes yielded similar genome size estimates of ∼720–730 Mb (Table 2).

**Table 2:** Properties of the *Q. lobata k*-mer distributions for *k* = 25 and *k* = 31

Word Size	k = 25	k = 31
Total *k*-mers	77,397,680,210	75,887,842,801
Error *k*-mers	1,753,595,327	2,535,065,256
Haploid coverage depth	51	49
Diploid coverage depth	106	101
Diploid genome size	720 Mb	730 Mb

Diploid genome size was estimated by dividing the number of *k*-mers under the haploid peak by haploid coverage depth, dividing all other *k*-mers counted by the diploid coverage depth, and summing these counts.

We separately estimated the haploid genome size using the assembly itself, which, as noted above, has a total size of 1.15 Gbp, much larger than *k*-mer estimate. An assembly much larger than the expected size usually occurs because of large numbers of uncollapsed haplotype variants; *i.e.*, regions of the *Quercus* genome where the two chromosomes differ sufficiently that the assembler creates two distinct contigs. However, we can identify these contigs based on depth of coverage: contigs representing haplotype variants will have approximately half the coverage of contigs representing homozygous regions. We computed the coverage of all contigs by remapping the reads to them, and plotted a coverage histogram, which yielded two distinct peaks (Figure 2B). In this plot, the lower coverage peak contains heterozygous regions for which the assembler created two contigs to represent the same chromosomal region. Thus, to estimate genome size, we halved the total contig length under the haploid peak, and added the result to the cumulative length of contigs in the right or “homozygous” peak. This estimated genome size from this method is ∼743 Mb, similar to the *k*-mer based estimates. We also used GenomeScope (http://qb.cshl.edu/genomescope/) to estimate the genome size from the complete set of corrected reads. This method gave an estimate of 702 Mb, and estimated heterozygosity at 1.25%, which would explain why so much of the assembly consisted of haplotype variants.

### BUSCO results

**Figure 3:**
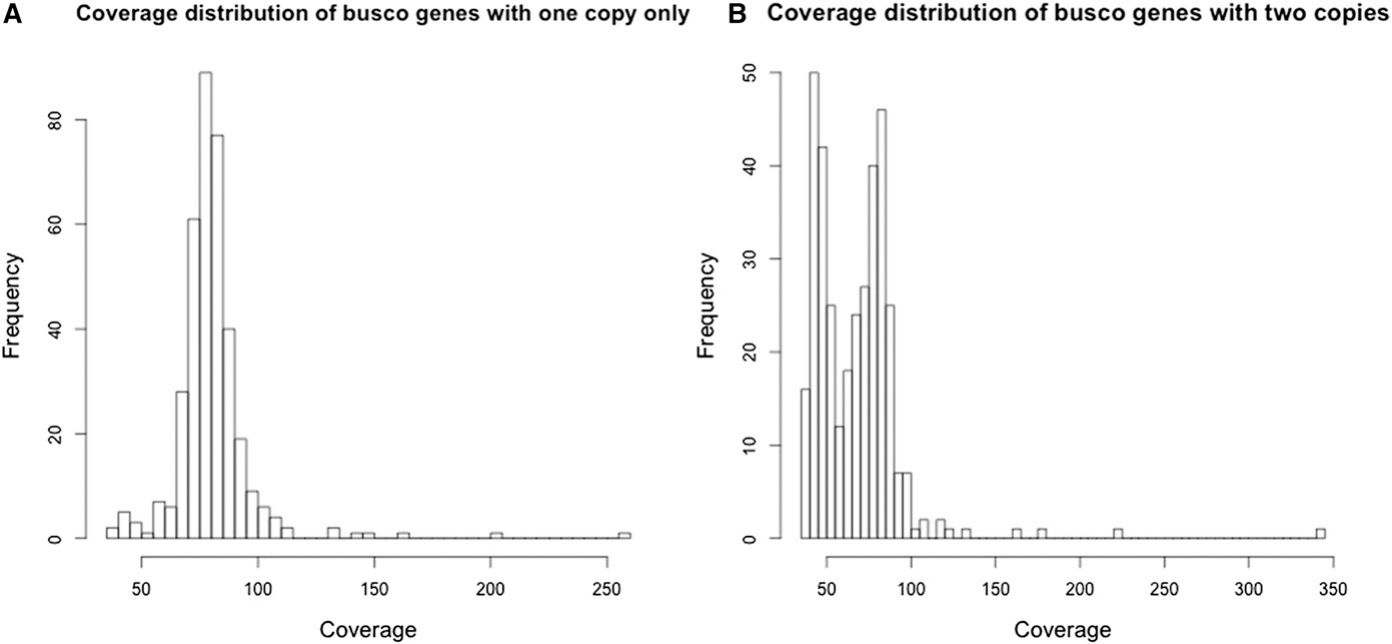
Average read coverage of BUSCO genes for *Q. lobata* v0.5 (collapsed to reduce haplotype duplication). We mapped 133 million pairs of reads to the assembled contigs, yielding an expected mean coverage of 75×. (A) BUSCO genes that are represented only once in the genome show a unimodal distribution around the expected coverage. (B) BUSCO genes represented twice in the genome show a bimodal distribution due to some genes having only half the expected coverage. These 0.5× coverage genes are presumably in genome regions for which the collapsing of haplotypes failed, leaving both haplotypes represented as independent contigs. The genes falling in the 1× coverage peak are expected to be truly present in two copies.

We used a BUSCO analysis to assess the completeness of the genome assembly and as another measure of the degree of haplotype separation. BUSCO databases are made from orthologous groups of genes present as single-copy orthologs in at least 90% of species (Simão *et al.* 2015). We used the plant database representing 956 ortholog groups. The BUSCO software identifies candidate gene regions, predicts gene structures, and then uses profile-based alignments to classify matches as complete, duplicated, fragmented, or missing. Table 3 summarizes the results of this analysis for the *Q. lobata* v1.0 genome as well as for the “collapsed” version, *Q. lobata* v0.5, the *Q. robur* genome (Plomion *et al.* 2016), and the well-studied Black Cottonwood tree, *P. trichocarpa* (Tuskan *et al.* 2006). Table 3 shows that 94% of the BUSCO orthologs were detected in our genome, with only 4% as fragmented genes. The *Q. robur* genome, built with sequences from four different platforms, including Sanger sequencing of BAC library ends, has only slightly higher coverage (97%). The model species, *P. trichocarpa*, is slightly higher again (98%). *Q. lobata* v1.0 and *Q. robur* both have high levels of duplication, indicating the frequent representation of both haplotypes in the genome due to the high heterozygosity. The rate of 50% duplication for *Q. lobata* v1.0 is consistent with the above contig coverage-based estimate of a true genome size of ∼730 Mb. The assembled genome size is 1.15 Mb, indicating 400 Mb, or ∼53%, is redundant. However, the collapsed version of the *Q. lobata* genome, and the well finished *P. trichocarpa* genome, still have 37% duplicates, which may be due to large-scale genome duplication events. The *P. trichocarpa* lineage is inferred to have undergone a whole genome duplication ∼60 million yr ago (MYA), with duplicates still detectable for >20% of its genes. Duplicates due to separated haplotypes are expected to have half the raw read coverage when compared to genes that are truly duplicated in the genome. Analysis of the raw read coverage for the *Q. lobata* v0.5 duplicated genes shows a bimodal distribution at 0.5 and 1× of the expected coverage, where coverage for single copy genes is a unimodal distribution at 1× (Figure 3). For the BUSCO genes with exactly two copies in the collapsed *Q. lobata* genome, > 60% are within the 1× coverage peak, suggesting > 50% are true duplicated genes rather than separated haplotypes. Similar to *P. trichocarpa*, and many plant lineages, this outcome may be due to a past whole genome duplication.

**Table 3:** Summary of BUSCO analysis indicating the number of BUSCO plant single copy orthologs detected in each of four genome assemblies: the *Q. lobata* genome reported here (v1.0), a collapsed version of the *Q. lobata* genome (v0.5), the European oak *Q. robur* (Plomion *et al.* 2016), and the black cottonwood tree *Populus trichocarpa* (Tuskan *et al.* 2006)

	*Q. lobata* v1.0	*Q. lobata* v.05	*Q. robur*	*P. trichocarpa*
Complete	863 (90%)	751 (79%)	885 (93%)	931 (97%)
Duplicated (% of complete)	450 (52%)	279 (37%)	437 (49%)	341 (37%)
Fragmented	35 (4%)	96 (10%)	29 (3%)	9 (1%)
Missing	58 (6%)	109 (11%)	42 (4%)	16 (2%)
Total BUSCO groups	956	956	956	956

### Annotation

**Figure 4:**
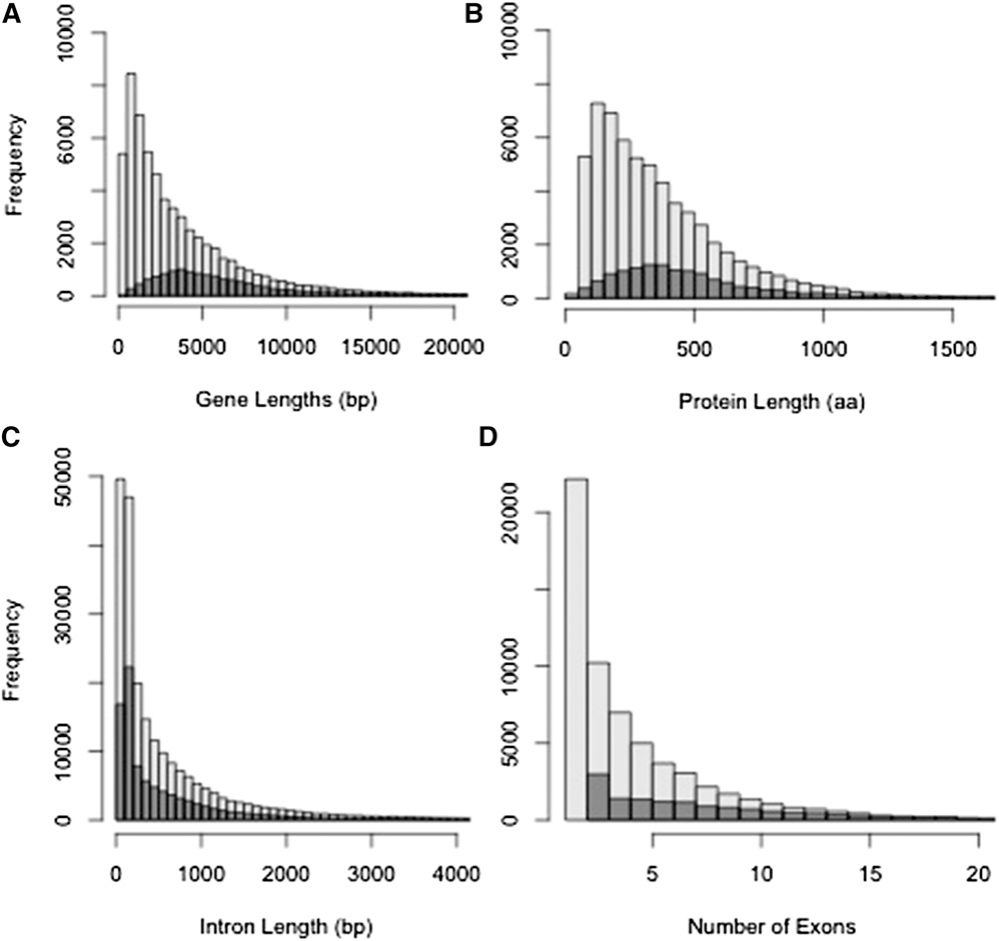
Frequency distribution results of Maker annotation of the *Q. lobata* v1.0 genome. (A) Transcript lengths. (B) Protein lengths. (C) Intron lengths. (D) Number of exons. Light gray bars represent all 61,773 gene models. Dark gray bars represent the high confidence subset of 13,898 models.

Using an automated pipeline based on Maker, we generated 61,773 gene models, of which 13,898 are high confidence, as defined in the *Materials and Methods*. For our full set of 61,773 gene models, 54,782 (89%) have an annotated estimated distance (AED) < 0.5 (Figure 4), and 43,706 (71%) have a recognizable PfamA domain. The annotations are of reasonable quality based on the rule of thumb presented by the Maker authors: “a genome annotation build where 90% of the annotations have an AED < 0.5, and over 50% of its proteome contains a recognizable domain can be considered well annotated” (Campbell *et al.* 2014). An AED of 0 means the extrinsic evidence (*e.g.*, protein and transcriptome alignments) perfectly matches the predicted model, and AED of 1 means there is no extrinsic evidence to support the model.

We compared metrics for the full set of gene models, and for the smaller high confidence set for transcript lengths (Figure 4A), protein lengths (Figure 4B), intron lengths (Figure 4C), and number of exons (Figure 4D). For the 13,898 high confidence models, the average predicted transcript is 6575 (SD = 5331) bases, has 8.0 (SD = 5.8) exons, an average intron size of 656 (SD= 990) bases, and codes for 491 (SD = 341) amino acids. However, only 62% of these high confidence gene models have both 5′ and 3′ predicted untranslated regions (UTRs). The average lengths of the predicted UTRs are 184 (SD = 258) bases for 5′, and 306 (SD = 319) bases for 3′ UTRs. Addition of the lower confidence gene models brings the average transcript length and number of exons down to 60% of the high confidence numbers (3969 bases and 4.8 exons). The protein length similarly goes down to 70% (375 amino acids).

The 61,773 predicted protein sequences were searched against the UniProt/Swiss-Prot database using blastp (cutoff 1 × 10^−6^) resulting in 41,612 (67%) with similarity to a UniProt/Swiss-Prot entry. Additionally, an InterProScan search resulted in 43,705 (71%) predicted proteins with similarity to a PfamA entry, and 25,536 (41%) were assigned GO terms.

Liftover of the 61,773 gene models from assembly version 1.0 to version 0.5 using a pipeline based on the UCSC-Kent toolkit, was successful for 71% of the models, giving 43,864 version 0.5 gene models. The decreased number is consistent with the previously discussed redundant haplotype variants in version 1.0, and the missing regions in version 0.5.

Known repeat elements present in the eudicotyledons subset of RepBase were identified for version 1.0 using RepeatMasker. Matches were found for 10.44% of the genome, of which 6.34% was to retroelements, 0.76% was to DNA transposons and 2.49% was to simple repeats (Table 4). Similar results were found for genome version 0.5 (data not shown).

**Table 4:** RepeatMasker results for genome version 1.0

	Number of Elements*^a^*	Length Occupied (bp)	Percentage of Sequence (%)
Retroelements	119,106	74,981,848	6.34
SINEs	1214	141,334	0.01
Penelope	0	0	0.00
LINEs	33,149	15,516,316	1.31
CRE/SLACS	0	0	0.00
L2/CR1/Rex	0	0	0.00
R1/LOA/Jockey	0	0	0.00
R2/R4/NeSL	0	0	0.00
RTE/Bov-B	2013	670,790	0.06
L1/CIN4	31,146	14,847,288	1.26
LTR elements	84,743	59,324,198	5.02
BEL/Pao	0	0	0.00
Ty1/Copia	23,614,490	35,709	2.00
Gypsy/DIRS1	31,606,882	40,559	2.67
Retroviral	0	0	0.00
DNA transposons	37,056	8,974,135	0.76
hobo-Activator	16,517	4,618,534	0.39
Tc1-IS630-Pogo	51	7850	0.00
En-Spm	0	0	0.00
MuDR-IS905	0	0	0.00
PiggyBac	0	0	0.00
Tourist/Harbinger	5412	1,226,849	0.10
Other (Mirage P-element, P-element, Transib)	0	0	0.00
Rolling-circles	0	0	0.00
Unclassified	4672	2,347,981	0.20
Total interspersed repeats		86,303,964	7.30
Small RNA	1455	312,636	0.03
Satellites	650	73,309	0.01
Simple repeats	787,721	29,398,846	2.49
Low complexity	148,512	7,684,148	0.65

All 94,394 contigs, total length 1,182,727,890 bp (1,069,186,757 bp excl *N*-runs) run with query eudicotyledons. GC level are 35.36%. 123,530,980 bp (10.44%) are masked.

a Most repeats fragmented by insertions or deletions have been counted as one element.

### Oak genome browser

**Figure 5:**
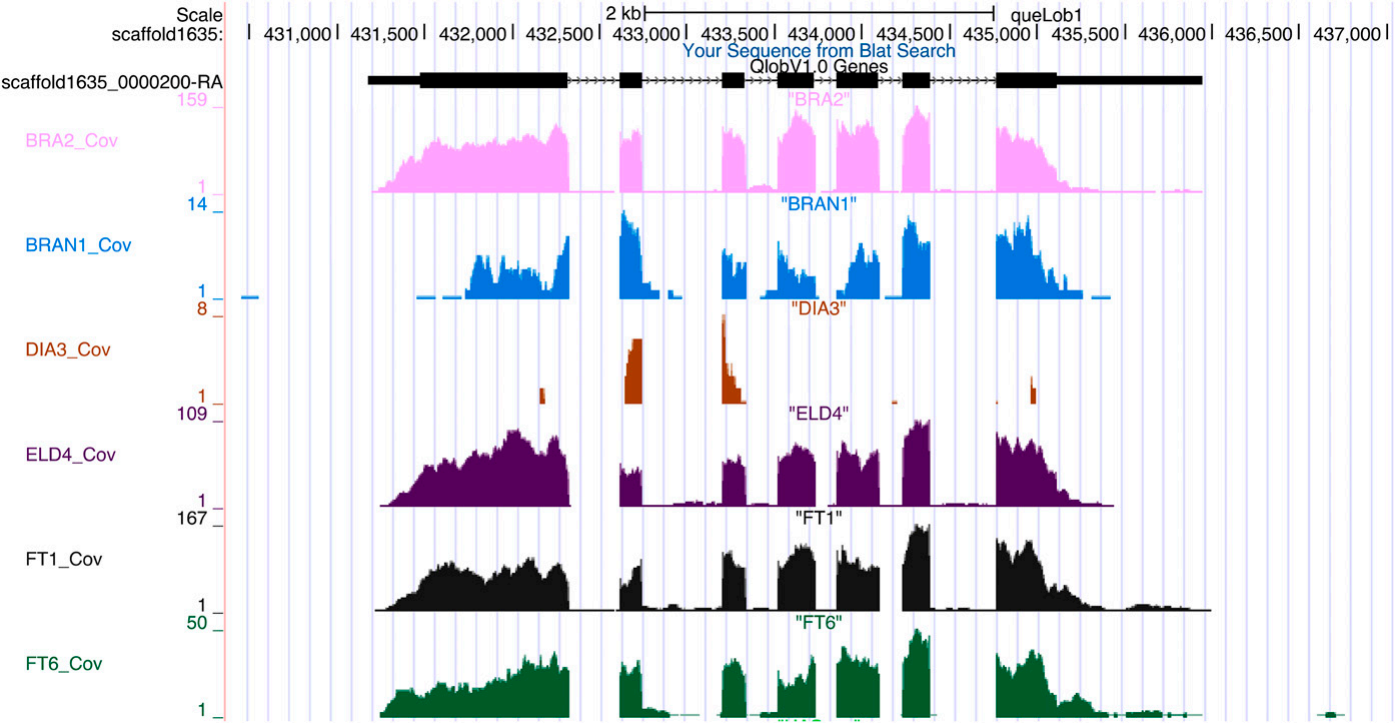
View of a cysteine-rich receptor-like protein kinase 29 gene in *Q. lobata*. The gene model is shown at the top, and below are the tracks for the expression of these gene across different trees, shown as counts of RNA-seq reads. The gene has a stress/antifungal domain, and exhibits low expression in one of the trees (DIA3), demonstrating that the expression of this locus is variable across trees (see https://valleyoak.ucla.edu/genomicresources/).

We have uploaded all data associated with our oak genome onto the University of California, Los Angeles (UCLA) instance of the UCSC Genome browser, which can be accessed at https://valleyoak.ucla.edu/genomicresources/. This provides data visualization capabilities to the community, as well as data import and export functions. Currently the site contains the genome sequence, the gene models and annotation described above, as well as RNA-seq data from a previous study (Cokus *et al.* 2015). We show a sample locus with corresponding gene models and RNA-seq data in Figure 5.

### Comparison with other oaks

We aligned the nuclear genome assembly of *Q. lobata* with the published *Q. robur* assembly using bwa-mem from the bwa package (Law and Jacobsen 2010). The *Q. robur* assembly comprises 17,910 scaffolds (> 2 kb) spanning 1.32 Gb. The additional length can be attributed to genomic heterozygosity. Overall the two species are very similar, with an average of 93.1% identity between the two assemblies. Despite the difference in length (1.32 *vs.* 1.15 Gb), 97% of each genome aligns to the other species, confirming that many of the scaffolds represent heterozygous variants that are very similar to other scaffolds within the assembly.

We scanned both assemblies for scaffolds that were unique to their respective genomes. These represent either insertions in one species, or regions that were missing from that species’ assembly. Considering only scaffolds of 2000 bp or longer, *Q. lobata* contains 1471 scaffolds for which all or part of the scaffold is not found in *Q. robur*. The total length of the missing regions is 4,853,580 bp (0.41%). For *Q. robur*, we found 1011 scaffolds with a total length of 2,989,698 bp (0.23%) that are not present in *Q. lobata*. Thus, only a tiny fraction of either genome is unique to that species.

We also compared the *Q. lobata* chloroplast with the closest sequenced relative, *Q. aliena* (Lu *et al.* 2015). The two assemblies are 98.93% identical. In addition to single nucleotide differences and small indels, the differences include two larger insertions in *Q. lobata* of 44 and 143 bases. In total, the *Q. aliena* assembly is 368 bases smaller than *Q. lobata*. We also compared the *Q. lobata* chloroplast to *Q. rubra*, which is in a different section of the genus *Quercus* (section *Lobatae*), but had no major indels, and is closer in size. The two chloroplast assemblies are 99% identical, but the *Q. lobata* is 15 bases smaller and differences consist entirely of single nucleotide substitutions and small indels. If only aligned sequence identity is taken into account, *Q. aliena* is 99.05% identical with *Q. lobata*, which is in agreement with the established relationship of *Q. lobata*, *Q. aliena*, and *Q. rubra*.

### Closing comments

The valley oak genome assembly here represents a first phase of the production of a high-quality, well-annotated genome. This first draft assembly indicates that the genome size is about 730 Mb, which is slightly smaller than estimated for *Q. robur* (Plomion *et al.* 2016), mostly likely due to differences in assembly methods rather than evolutionary histories. Because of the high heterozygosity in this high-outcrossing genus, duplication of haplotypes easily leads to overestimates of genome size, which motivated us to include here a genome version that was modified to reduce haplotype duplication (v0.5: https://valleyoak.ucla.edu/genomicresources/). This reduced version has allowed us to more effectively call SNPs in other studies. For example, we have already used v0.5 for an epigenetic study of valley oak where we were able to identify some genes with significantly high methylation levels that were correlated with a climate variable (Gugger *et al.* 2016b). We have several landscape genomic across several species of oak, and phylogenetic studies are in progress using this collapsed version. In addition, we are in the process of manually improving the annotations for genes in v1.0, especially for those associated with response to climate environments, such as bud burst and drought response. Future developments of the valley oak genome underway through PacBio sequencing and improvements in annotation will facilitate research in comparative genomics, macroevolution, and phylogenetics in *Quercus* and Fagaceae. Given the ecological and economic status of oaks, and the relative small size of this genome, oak genomic research should provide valuable case studies for evolutionary questions about genes and genic regions involved in the adaptation, hybridization, and epigenetics that help shape the response of trees to their environment.
